# Acute and short-term administrations of delta-9-tetrahydrocannabinol modulate major gut metabolomic regulatory pathways in C57BL/6 mice

**DOI:** 10.1038/s41598-019-46478-0

**Published:** 2019-07-19

**Authors:** Megha Oza, William Becker, Phani M. Gummadidala, Travis Dias, Mayomi H. Omebeyinje, Li Chen, Chandrani Mitra, Rubaiya Jesmin, Paramita Chakraborty, Mathew Sajish, Lorne J. Hofseth, Koyeli Banerjee, Qian Wang, Peter D. R. Moeller, Mitzi Nagarkatti, Prakash Nagarkatti, Anindya Chanda

**Affiliations:** 1 Environmental Health Sciences, Arnold School of Public Health, University of South Carolina, Columbia, SC USA; 2 Department of Pathology, Microbiology, and Immunology, School of Medicine, University of South Carolina, Columbia, SC USA; 3 Creative Proteomics Inc., Shirley, New York USA; 4 Department of Statistics, University of South Carolina, Columbia, SC USA; 5 Drug Discovery and Biomedical Sciences, College of Pharmacy, University of South Carolina, Columbia, SC USA; 6 National Institutes of Health, Bethesda, MD USA; 7 Department of Chemistry and Biochemistry, University of South Carolina, Columbia, SC USA; 8 National Ocean Service, Hollings Marine Laboratory, Charleston, SC USA

**Keywords:** Metabolomics, Metabolomics

## Abstract

Delta-9-tetrahydrocannabinol (THC) is the primary psychoactive compound in Cannabis, which is studied extensively for its medicinal value. A central gap in the science is the underlying mechanisms surrounding THC’s therapeutic effects and the role of gut metabolite profiles. Using a mass-spectrometry based metabolomics, we show here that intraperitoneal injection of THC in C57BL/6 mice modulates metabolic profiles that have previously been identified as integral to health. Specifically, we investigated the effects of acute (single THC injection denoted here as ‘1X’) and short -term (five THC injections on alternate days denoted as ‘5X’) THC administration on fecal and intestinal tissue metabolite profiles. Results are consistent with the hypothesis that THC administration alters host metabolism by targeting two prominent lipid metabolism pathways: glycerophospholipid metabolism and fatty acid biosynthesis.

## Introduction

The medical use of Cannabis (commonly termed ‘marijuana’), a product from the plant, *Cannabis sativa*, is becoming increasingly popular worldwide for its medicinal value^1–3^. In the USA, 33 states and District of Columbia have already legalized the medical use of marijuana^4^. Delta-9-tetrahydrocannabinol (THC) that was first described in 1964 is the primary psychoactive compound in Cannabis and is known to display therapeutic potentials as an analgesic, antiemetic and appetite stimulant^2,3,5–8^. Additionally, THC can be used for the treatment of multiple acute and chronic health disorders^9–11^. These include treatment of nausea and vomiting associated with cancer chemotherapy, anorexia, and cachexia associated with HIV and AIDS patients, pain and muscle spasms in multiple sclerosis^12^. Their anti-inflammatory effects have been tested in experimental models for autoimmune disorders such as multiple sclerosis, rheumatoid arthritis, colitis, hepatitis and cancer^9^. Importantly, during the past several years, THC content in marijuana has been steadily increasing^13,14^; and recreational use has expanded^15^. Hence, the scientific premise for studying the mechanisms involved in the modulation of disease by THC is strong.

It is well established that THC can be useful in preventing and ameliorating the symptoms of intestinal inflammation such as abdominal pain, diarrhea and reduced appetite in patients suffering from inflammatory bowel disease (IBD)^16–19^. In animal models of colitis, THC demonstrated successful reduction of 2,4,6-Trinitrobenzene sulphonic acid-induced mucosal damage of the intestine, neutrophil infiltration and *in vitro* motility disturbances. It has been shown^20–22^ that the epithelial wound healing, inhibition of pro-inflammatory cytokine and chemokine release, immune cell recruitment, are mediated through activation of cannabinoid receptors (CB1 and CB2) of the endogenous cannabinoid system. Activation of these receptors is critical in the neuromodulatory actions on the sensory and autonomic nervous system connected with the pharmacological function of THC^23^.

Despite such well-documented evidence supporting THC’s therapeutic impact in safeguarding the healthy functioning of the intestine, the entire mechanism underlying the intestinal protection and other pharmacological effects of THC still remains unclear. This gap in understanding is at least in part due to the absence of knowledge on whether THC influences host metabolism and how. The knowledge of the key metabolic targets of THC is critical for appropriate therapeutic applications of THC. Hence the main objective of this study was to investigate the influence of acute and short-term THC administration on fecal and intestinal tissue metabolome of wild-type C57BL/6 mice. A comparative untargeted metabolome profiling of fecal and intestinal tissue samples obtained from THC administered mice and control mice that received vehicle instead, was conducted using an ultra-performance liquid chromatography-time of flight-mass spectrometry (UPLC-TOF-MS). Presented in this report are the results of this investigation.

## Materials and Methods

### Experimental animals, diet and THC administration

Female C57BL/6 (BL6) mice, aged 8–10 weeks, obtained from Jackson Laboratories were used for this study. All mice were housed in pathogen-free conditions and allowed *ad libitum* access to filtered water and Teklad rodent diet 8604 (regular chow) at the Animal Research Facility located at the University of South Carolina School of Medicine. To understand the effect of THC on gut metabolites we have followed our previously published protocols of THC administration^24–28^, whereby we injected 20 mg/Kg THC intraperitoneally. The experimental group (n = 5) received THC every 48 h. THC was dissolved in a vehicle of 100% ethanol, and both treatment and vehicle were administered in 100 µL of a combination of ethanol, Tween-80, and saline, at a ratio of 2:1:17. Animals were regularly monitored during the period of the experiment for any body weight changes, signs of toxicity and mortality. Fecal samples analyzed for comparative metabolomics were collected 24 h after the first administration (denoted here as 1X) and 24 h after the 5^th^ administration (denoted here as 5X). The 1X samples and 5X samples were used to study respectively, the acute and short-term effects of THC administration.

The rationale for using an intraperitoneal mode of exposure was the fast bioavailability of THC in the bloodstream, due to which we have consistently used this model^25–27^ as the closest intravenous self-administration paradigm because THC is unable to sustain in rodents upon intravenous administration^29^. As in our previous studies^25–27^, we reasoned here as well, that since i.p. administration would allow the THC to directly diffuse across the peritoneal membrane to the blood vessels of the abdominal viscera, musculature and mesentery, it would help in avoiding any possible artifacts resulting differential nutrient absorption rates caused by oral administration of THC.

The dose of THC in this study and our previous studies was determined according to body surface area normalization based calculations described earlier by Reagan-Shaw *et al*.^30^. Based on these calculations, our applied 20 mg/kg THC dose translates to 60 mg/m^2^ in humans, which is well within the maximum human recommended dose (MHRD) of synthetic THC of 90 mg/m^2^/day. We point out here that THC (also termed Dronabinol) has consistently been used for clinical use to reduce neuropathic pain in multiple sclerosis patients^31–33^. No teratogenic effects was reported in mice administered THC at up to 30 times the MRHD and up to 5 times the MRHD for patients with AIDS and cancer, respectively (see FDA data^34^). We have chosen mice of a single gender for our experiments to rule out any previously reported sex difference effects on THC metabolism^35,36^. Given that the prevalence of multiple sclerosis is about two to three times higher in women than men^37^, we have chosen female mice for this study.

### Ethics statement

The mice employed in this study were housed at the American Association for the Accreditation of Laboratory Animal Care (AAALAC)-accredited Animal Resource Facility at the University of South Carolina, School of Medicine, Columbia, SC. All experimental procedures were performed according to National Institutes of Health (NIH) guidelines under protocols approved by the University of South Carolina Institutional Animal Care and Use Committee.

### Sample preparation for metabolome analysis

Fecal samples were collected at the indicated time points by placing mice in individual cages with very little bedding but *ad libitum* access to food and water. Fecal pellets were immediately collected and placed on ice until a full sample was collected at which point the samples were immediately frozen in liquid nitrogen and transferred to −80. In order to ensure that only fresh feces were used for fecal metabolome analysis, the cages were changed every day. Fecal samples (0.1 g) from THC administered animals and controls were ultrasonically homogenized in 1 mL cold methanol/water (1:1) in for 30 mins followed by vortexing on cooled (4 °C) mixer for five mins. The homogenized samples were centrifuged for ten mins at 10000 × g at 4 °C. The supernatant (300 μL) was dried in a vacuum concentrator. The dry residue was re-dissolved in 150 μL methanol/water (1:1) before analysis. For intestinal tissues, the metabolites were extracted from 50 mg of lyophilized tissue samples with 800 µL of methanol. The samples were ground to fine powder using Grinding Mill at 65 Hz for 45 s. The ground samples were vortexed for 30 s, and centrifuged at 10,000 × g at 4 °C for 15 mins. Finally, 200 µL of supernatant was transferred to vial for LC-MS analysis.

### UPLC-ESI-QTOF-MS profiling of fecal metabolites

Metabolites were separated from injected samples (5 μL aliquots) using Ultra Performance Liquid Chromatograph (1290 Infinity Binary LC System, Agilent Technologies, USA) and screened with ESI-MS (targeted MS/MS mode). The chromatograph system comprised of Waters ACQUITY UPLC HSS T3 (100 × 2.1 mm,1.8 um) with Phenomenex Security GuardTM ULTRA. The mobile phase consisted of 0.1% formic acid-water (solvent A) and 0.1% formic acid-acetonitrile (solvent B) with a gradient elution (0−1 min, 95% A; 1−6 min, 95−70% A; 6−20 min, 70−5% A). The flow rate of the mobile phase was set at 0.5 mL·min-1. The column temperature was maintained at 45 °C, and the sample manager temperature was set at 4 °C.

Mass spectrometry was performed on Quadrupole/Time-Of-Flight Mass Spectrometer (QTOF-MS; model G6540B Agilent Technologies, USA) using a Dual Agilent Jet Stream (AJS) ESI source. Spectra were recorded in the scanning mass-to-charge (m/z) range of 50 to 1500 with a scan rate of 1.00 spectra·sec-1. The capillary voltage was set to 4000 V, and 3500 V (positive and negative mode, respectively) and the fragmentor was set to 175 V. The pressure of the nebulizer was set at 35 psi, the gas temperature to 325 °C, and the continuous gas flow to 5 L·min-1. The instrument mode was set to an extended dynamic range. Quality control was maintained by injecting a control sample after analysis of every ten samples. The needle was washed (3X) with 50% methanol before every injection to avoid cross-contamination of samples. A volume of 20 μL of methanol was injected for rinsing.

### UPLC-ESI-QTOF-MS profiling of intestinal tissue metabolites

Separation of the tissue metabolites was performed using a similar UPLC setup with the following modifications. The chromatography system comprised of an Agilent 959758-902 RRHD Eclipse Plus C18 (100 × 2.1 mm, 1.8 µm) with Phenomenex Security GuardTM ULTRA. The gradient elution was set to 0–1 min 95% A, 1–6 min 95–80% A, 6–9 min 80–50% A; 9–13 min 50–5% A; 13–15 min 5% A. The flow rate of the mobile phase was set at 0.35 mL·min^−1^. The column temperature was maintained at 40 °C, and the sample manager temperature is set at 4 °C. For mass spectrometry the spectra were recorded in the scanning mass-to-charge (m/z) range of 50 to 1000 with a inter scan time of 0.02 s. The capillary voltage was set to 4000 V and 3500 V (positive and negative mode, respectively) and the sampling cone was set to 35 kV and 50 kV (positive and negative mode, respectively). The cone gas flow was set to 50 L/h, the source temperature to 100 °C, and the extraction cone to 4 V. The desolvation temperature was set to 350 °C and 300 °C (positive and negative mode, respectively), and the desolvation gas flow is set to 600 L/h and 700 L/h (positive and negative mode, respectively).

### Metabolite data processing and analysis

For metabolite data processing, the acquired raw data were aligned using Mass Hunter Workstation (B0.06.00, Agilent) based on the m/z value and the retention time of the ion signals. Ions from both ESI− and ESI+ were merged into the SIMCA-P program (version 14.1) for multivariate analysis. The data were stored in a table with one sample per row and one variable (bin/peak/metabolite) per column. The ion intensities for each peak detected were then normalized to the sum of the peak intensities in the sample (SI Fig. 1), finally rendering a multi-dimensional dataset, comprising of a peak number based on the m/z value and the retention time of the ion signals and ion intensities. This dataset was used for multivariate data analysis (MetaboAnalyst 3.0.), which included Univariate Analysis, Principal Component Analysis (PCA) and Partial Least Squares - Discriminant Analysis (PLS-DA). The univariate analysis was used for exploratory data analysis to determine Fold Change (FC) of metabolites between experimental and control groups and conduct t-tests. The unsupervised PCA was used to visualize the variance in a data set per group and the separation between the experimental and the control groups. The supervised PLS-DA was used to assign the class to the metabolites determine the difference between the groups for each class. Finally, the variable importance of projection values (the VIP values) was computed from the weighted sum of squares of the PLS loadings considering the amount of explained permuted class level variation in each dimension.

### Identification of potential biomarkers and interpretation of metabolic signatures

The chemical structures of metabolites were identified according to online databases such as the Human Metabolome Database (www.hmdb.ca), Metlin (www.metlin.scripps.edu) and the Mass Bank (www.massbank.jp) using the data of accurate masses and MS/MS fragments. When necessary, further confirmation was acquired through comparisons with authentic standards, including retention times and MS/MS fragmentation patterns. To interpret the biological significance of the metabolic signatures, the metabolites that displayed significant differences between THC administered and control groups were imported to MBRole, a freely available web server for functional enrichment analysis on metabolic data from any organism^38,39^. Since a mouse model was used for this current study, the MBRole analysis was performed using the *Mus musculus* background.

### Sample size determination and statistical analyses

We used power analysis to determine the ideal sample size for our experiments. With the assumption of a normal distribution, a 20% change in mean and 15% variation in THC effect on gut metabolome, we determined that a sample size ≥4 would be required per group to surpass 80% power for the study, given that we used concurrent controls for the study. Hence, have used 5 animals per group. Metabolites that showed significant differences between THC administered and control (vector administered) groups were identified by combining the results of students t-test (p < 0.05), fold change (FC > 2) and variable importance in projection values (VIP > 1). To address the cases when the quantified metabolites fail to satisfy the normality and equality of variance based on Kolmogorov-Smirnov test and Levene’s test respectively, we used the non-parametric Kruskal-Wallis test to determine metabolite differences between the THC-administered and the vector-control groups. The acquired p-values were corrected for multiple testing using Benjamini and Hochberg False Discovery Rate (FDR)^40^, a method applied previously for untargeted metabolomic analysis^41^.

## Results

### Analysis of fecal metabolite profile shifts upon acute THC administration

**Figure 1:**
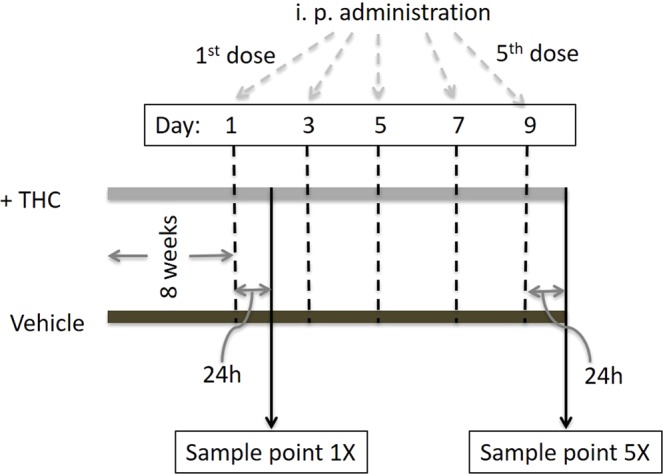
Study design. Fecal samples (n = 5) for analyses were collected at 2 time-points, 1X and 5X. The injections of THC and vehicle were administered intraperitoneally ~48 h apart (denoted dashed lines) Five administrations were conducted. Fecal and intestinal tissue samples were collected at time points 1X and 5X, which were 24 h after the first and the fifth administrations respectively.

**Figure 2:**
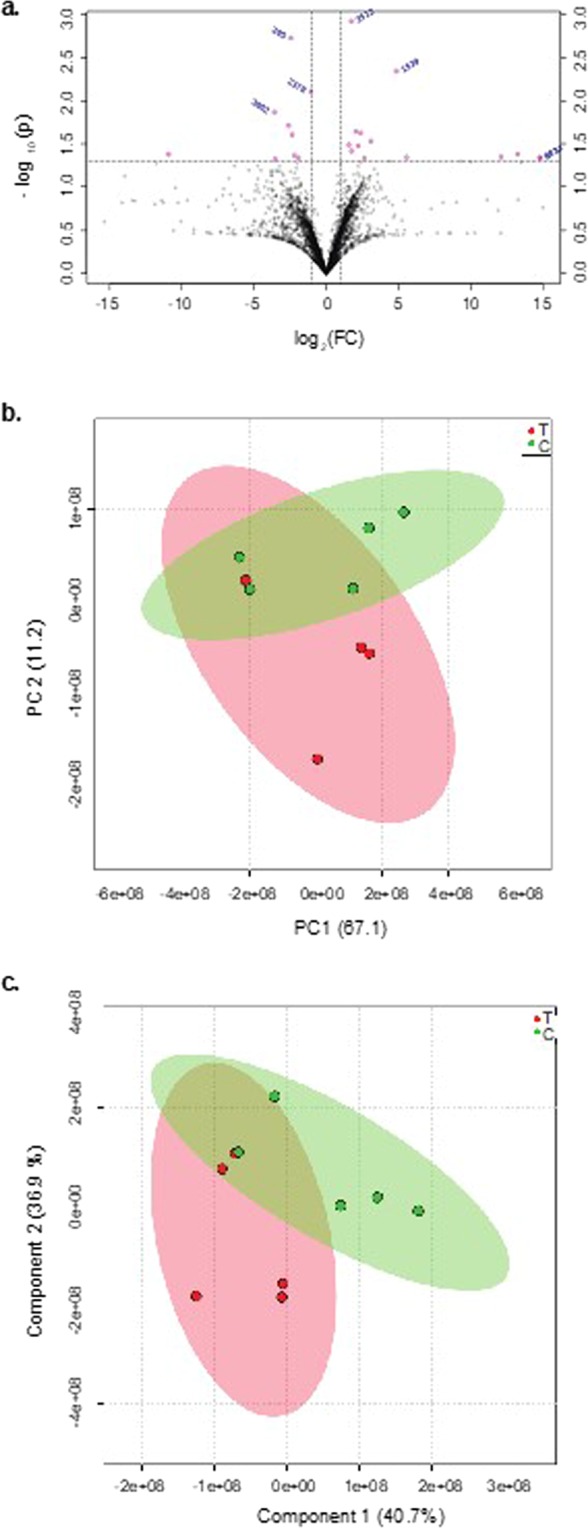
Fecal metabolome changes in 1X samples. (**a**) Volcano plots enabling the visualization of metabolites showing differential abundance. These were selected based on fold change (X-axis) and p-value in (Y-axis). The m/z values (highlighted in pink) represent a fold change of ≥0.5 or ≤2.0 and p-value ≤ 0.05 in THC administered mice compared to the vehicle controls and were selected for further characterization. (**b**) PCA score scatter plots based on fecal metabolic profiling of THC (n = 5) and control (n = 5) mice c) PLS- DA score plots based on detected fecal metabolites from THC administered (n = 5) and control (n = 5) mice. T, THC administered mice, C, control mice.

The experimental design of the experiment is illustrated in Fig. 1. To understand the acute effects of THC administration on gut metabolite profiles in C57BL/6 mice, we compared their fecal metabolite profiles 24 h after THC administration (sampling time point denoted here as ‘1X’). The results from our initial examination of base peak intensity chromatographs (SI Fig. 2) are shown as volcano plots in Fig. 2a, which indicated a shift in the profiles upon THC administration. An unsupervised evaluation of metabolic signatures was conducted using the indices PCA1 and PCA2 that were obtained upon PCA analysis by reduction of the multi-dimensional datasets to optimized and comparable datasets. As shown in Fig. 2b, the PCA scores scatter plot demonstrates a clear separation between the THC-treated and control fecal samples. Following PCA analysis, the identification of differential metabolites was performed using supervised partial least square- discriminant analysis (PLS-DA) on the MS data to predict the class membership and assess the significance of class discrimination. As shown in Fig. 2c, the PLS-DA scores scatter plot suggested that THC administration again demonstrates a significant class separation between the THC-treated and control fecal samples.

### Analysis of fecal metabolite profile shifts upon short-term THC administration

**Figure 3:**
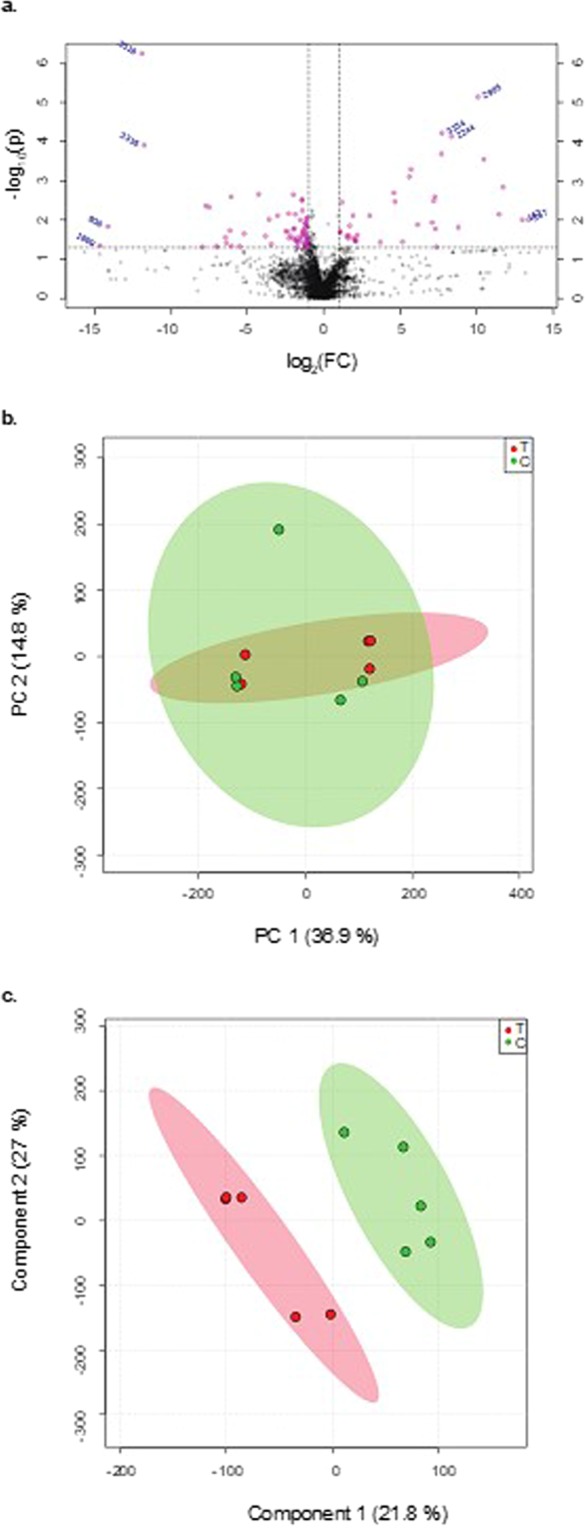
Fecal metabolome changes in 5X samples. (**a**) Volcano plots showing the metabolites with differential abundance between THC treated mice and the controls. These were selected based on fold change (X-axis) and p-value in (Y-axis). The m/z values (highlighted in pink) represent a fold change of ≥0.5 or ≤2.0 and p-value ≤ 0.05 in THC administered mice compared to the vehicle controls and were selected for further characterization. (**b**) PCA score scatter plots based on fecal metabolic profiling of THC (n = 5) and control (n = 5) mice c) PLS- DA score plots based on detected fecal metabolites from THC administered (n = 5) and control (n = 5) mice. T, THC administered mice, C, control mice.

To understand the short-term effects of THC administration on gut metabolite profiles in C57BL/6 mice, we compared fecal metabolite profiles 24 h after five THC injections(sample denoted here as ‘5X’). The volcano plot (Fig. 3a) summarizing our initial examination of the fecal metabolites indicates a shift in fecal metabolite profiles with this multiple exposure protocol (base peak intensity chromatographs of fecal metabolites of 5x are shown in SI Fig. 2). The separation between the fecal metabolite profiles of the THC administered and control groups was further confirmed by the PCA scores scatter plot (Fig. 3b). Finally, the supervised PLS-DA scores obtained from the MS data was used to predict the class membership and demonstrate the significance of class discrimination (Fig. 3c).

### Identification of potential biomarkers

From the comparisons of fecal metabolite profiles between THC administered and the control groups, a group of ‘significant metabolites’ was identified using the criteria of VIP value > 1. The higher VIP values were indicative of a higher contribution from these metabolites toward the differential profiles between the THC and the control groups. A list of these metabolites from the comparisons of 1x with their controls and the 5x with their controls is provided in Table 1 a and b. Along with the VIP values for each metabolite, Table 1 also indicates the fold change of the increase or decrease in the metabolite concentration upon THC administration. The list of identified significant metabolites that were identified differentially upon THC administration was entirely different between samples 1X and 5X, suggesting that acute and short-term administrations have different functional impacts on the mouse gut.

**Table 1:** Fecal metabolites that demonstrated significant changes upon THC administration.

Metabolite Name (*§*)	Associated metabolic process (*)	Molecular Mass	+/−	Fold Change	Adjusted P value
**Fecal metabolites showing differential abundance in 1X samples**					
PE(18:3(6Z,9Z,12Z)/0:0)	Glycerophospholipid metabolism	474.2582	+	550.97	0.0049
Methylxanthine	Caffeine metabolism	189.0431	+	2959.7	0.006
DG(22:5(4Z,7Z,10Z,13Z,16Z)/20:3(5Z,8Z,11Z)/0:0)	Glycerophospholipid metabolism	691.5149	+	2793.4	0.006
PS(20:5(5Z,8Z,11Z,14Z,17Z)/20:1(11Z))	Glycerophospholipid metabolism	834.5193	+	2729.4	0.006
N-Acetylcarbocysteine	Unknown	220.0348	+	351.89	0.006
6-Hydroxymelatonin	Tryptophan metabolism	249.1269	+	618.81	0.0272
PG(20:4/0:0)	Glycerophospholipid metabolism	531.287	+	987.45	0.0356
Asp Ser Gln	Unknown (possibly endogenous opioid peptide synthesis)	347.1231	+	63.882	0.037
Pro Tyr Val	Unknown (possibly endogenous opioid peptides synthesis)	376.1897	+	850.9	0.045
PS(O-16:0/13:0)	Glycerophospholipid metabolism	678.4848	−	1470.5	0.003
S-Prenyl-L-cysteine	Unknown	187.0634	−	8333.3	0.005
PC(6:0/6:0)	Glycerophospholipid metabolism	424.2828	−	400	0.006
2E-methyl-glutaconic acid	Valine, Leucine, Isoleucine degradation	145.0528	−	909.1	0.007
Tiglylglycine	Isoleucine degradation	158.0826	−	500	0.011
2-Methyleneglutarate	Nicotinate and Nicotinamide metabolism	145.0527	−	20	0.045
**Fecal Metabolites showing differential abundance in 5X samples**					
**Metabolite Name** (*§*)	Associated Metabolic process (*)	**M/Z**	**+/−**	**Fold Change**	**Adjusted P Value**
Sphingosine	Sphingolipid metabolism	317.3092	**+**	35.5	0.04
2S-Hydroxytetradecanoic acid	Fatty acid biosynthesis	244.370	**+**	8202.6	0.009
Beta-Hydroxy palmitic acid	Fatty acid biosynthesis	272.429	**+**	10835	0.01
Dodecanamide	Fatty acid biosynthesis	217.2332	**+**	2.8	0.03
1-Phenyl-1,3-eicosanedione	Unknown	387.3372	**+**	70.286	0.01
N-stearoyl taurine	Taurine and Hypotaurine metabolism	390.2781	**+**	445.28	0.02
2-Hydroxyenterodiol	Unknown	336.1884	**−**	200	0.005
Hydroxymalonate	Unknown	138.0423	**−**	4.4	0.03
PA(17:2/22:2)	Lipid metabolism	797.5431	**−**	3333.3	0.0001

(*§*) Putative Metabolites were identified through interpretation of tandem MS data. Feature identities were determined using a two-step approach described previously^76^: (1) search of public databases including METLIN, HMDB and Mass Bank using accurate mass and a mass error window of 10 ppm and (2) comparison of tandem MS data with available spectra for four important features followed by manual interpretation.

(*) The metabolic processes indicated in this column are the processes that most closely associated with the detected metabolites. This means that the detected metabolites may be an intermediate, a product or derivatives of intermediates or products of the mentioned processes. The metabolic processes were identified based on available information in HMDB, Kegg database and Lipidomics Gateway. The compounds for which we could not find any information are denoted as ‘Unknown’.

(+/−) Increase/Decrease.

### Interpretation of metabolic signatures

**Figure 4:**
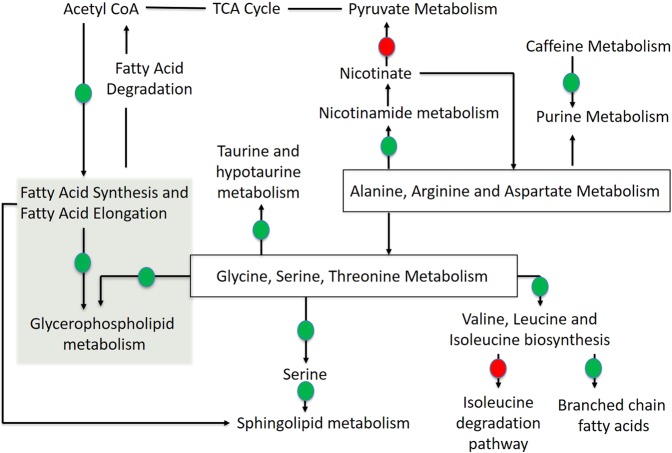
Functional pathway analysis showing the major pathways that are upregulated or downregulated upon administration of THC. Biological relationships of the pathways were adapted from KEGG, Lipid Maps and HMDB database based on the identification of the metabolite markers corresponding to the pathways. Based on number of identified metabolites with >2 fold increase/decrease, we indicate the processes that are upregulated with green circles and the processes that are downregulated with red circles. The green highlighted zone show the metabolic pathways for which metabolite enrichment was observed both in fecal and intestinal tissue metabolite profiling.

To investigate the latent relationships of the differential metabolites listed in Table 1 and gain insights into metabolite enrichment representing specific metabolic pathway and molecular network perturbations induced by THC exposure, the information of the significant metabolites was imported to the MBRole platform. The enrichment analysis of the metabolic signatures was performed on *Mus musculus* background using annotations from the KEGG^42^ and HMDB databases^43^. While the former database primarily annotates metabolites with their associated pathways and enzymes, the later annotates metabolites with diseases, pathways, tissues, biofluids and the cellular localization. Figure 4 summarizes our observations from this enrichment analysis and indicates the potential metabolic processes that are impacted by THC administration, which in turn, could help explain its therapeutic impact on many diseases. Specifically, acute administration of THC demonstrated a positive correlation with the metabolic intermediates of Glycerolipid metabolism, PI3K/AKT/mTOR and lysophopholipid signaling, opioid peptide biosynthesis and endocannabinoid signaling. Also, the acute administration was negatively correlated with the metabolite intermediates of nicotinate degradation. Nicotinate being the precursor for the generation of nicotinamide adenine nucleotide (NAD^+^)^44^, our study suggests a positive effect of THC on cellular NAD^+^ levels and upregulation of systemic NAD^+^ is demonstrated to have profound health beneficial effects^45–47^. Short-term administration of THC was positively correlated with the fatty acid degradation pathway, sphingosine metabolism, caffeine metabolism as well as endocannabinoid signaling. Additionally, the short-term THC administration was negatively correlated with xenobiotic metabolism. These processes have significant cross-talks with several important metabolic processes such as amino acid, carbohydrate, and nucleotide metabolism. Specifically, the glutathione metabolism and branched chain amino acid (BCAA) pathways are highly enriched. Interestingly, dysregulation of these metabolic pathways are known to be associated with the etiology of diabetes, obesity, cancer, and neurodegeneration^48–50^. Our future work will investigate the specific metabolite changes in various preclinical models such as mice models for acute and short-term intestinal inflammation. Those studies will further elucidate the mechanistic details of the THC-mediated health benefits.

### Metabolite profiling of intestinal tissues: validation of fecal metabolite profiles

Finally, to determine whether our metabolome analysis of fecal samples represent changes in host metabolism induced by THC administration, we performed similar untargeted metabolite profiling intestinal tissues obtained from 1X and 5X THC administered mice and the corresponding vector controls. The list of ‘significant metabolites’ identified from these comparisons using the criteria of FC > 2, VIP > 1 and adjusted P-value < 0.05 are shown in Table 2; associated volcano plots, PCA plots and the PLS-DA plots are included as supplementary Information (see SI Fig. 3). Also outlined in Table 2 are the potential metabolic processes that the identified metabolites associate with. Lipid metabolism, especially glycerophospholipid metabolism and fatty acid biosynthesis, emerged as the global metabolic process that was most significantly influenced by THC administration. As shown in Fig. 4, lipid metabolism is a core host metabolic process within the global host metabolic network. Results are consistent with the hypothesis that the altered fecal metabolite profiles seen upon 1X and 5X THC administrations, at least in part, were reflective of the THC-mediated alteration in host metabolism.

**Table 2:** Metabolites showing differential abundance in 1X and 5X intestinal tissue samples.

Metabolite Name *(§)*	Associated metabolic process	M/Z	1X			5X		
			+/−	Fold change	p-value (adj.)	+/−	Fold change	p-value (adj.)
N- Heptanoylglycine	Fatty acid metabolism	186.11	**+**	**4.7**	0.003			
N-Decanoyl glycine	Fatty acid metabolism	228.16	**+**	4.8	0.04			
2-hydroxy-6-methoxy-4-(prop-2-en-1-yl)phenyl]oxidanesulfonic acid	Unknown	259.03	**+**	2.1	0.01			
Inosine	Purine metabolism	267.03	**+**	2.2	0.01			
Pe-nme2(14:0/18:1(11Z))	Glycerophospholipid metabolism	716.52	**−**	2.3	0.0006			
(2-Aminoethoxy)[(2R)-3-[(1Z,9Z)-octadeca-1,9-dien-1-yloxy]-2-[(9Z,12Z,15Z)-octadeca-9,12,15-trienoyloxy]propoxy]phosphinic acid	Glycerophospholipid metabolism	722.51				**+**	4.1	0.006
PE(P-16:0/22:6(4Z,7Z,10Z,13Z,16Z,19Z))	Glycerophospholipid metabolism	746.51				**+**	2.5	0.02
(2-Aminoethoxy)[(2 R)-3-[(7Z,10Z,13Z,16Z)-docosa-4,7,10,13,16-pentaenoyloxy]-2-[(1Z)-hexadec-1-en-1-yloxy]propoxy]phosphinic acid	Glycerophospholipid metabolism	748.53				**+**	3.1	0.0008
PG(18:0/18:2(9Z,12Z))	Glycerophospholipid metabolism	774.54				**+**	2.4	0.03
PG(18:0/18:1(9Z))	Glycerophospholipid metabolism	775.54	**−**	2.6	0.008			
PS(15:0/20:0)	Glycerophospholipid metabolism	776.55				**+**	4.4	0.04
PS(15:0/22:0)	Glycerophospholipid metabolism	804.57				**+**	3.6	0.03
PA(22:6(4Z,7Z,10Z,13Z,16Z,19Z)/24:1(15Z))	Glycerophospholipid metabolism	829.57			0.02	**+**	7.3	0.03
Palmitamide	Fatty acid biosynthesis	256.26	**+**	3.1	0.01			
Palmitic acid	Fatty acid biosynthesis	257.27	**+**	3.0	0.01			
5-[3,5-Bis(butan-2-yl)cyclopent-1-en-1-yl]-5-hydroxy-3-oxopentanoic acid	Fatty acid biosynthesis	311.23	**+**	3.7	0.002			
1,2,4-Nonadecanetriol	Fatty acid biosynthesis	317.30	**+**	2.5	0.001			
Docosanamide	Fatty acid biosynthesis	340.36				**−**	5.2	0.01
1,3-Dihydroxypropan-2-yl (5Z,8Z,11Z)-icosa-5,8,11-trienoate	Fatty acid biosynthesis	381.30	**+**	9.1	0.01			
12alpha-hydroxy-3-oxo-5beta-cholan-24-oic acid	Fatty acid biosynthesis	391.28				**+**	3.8	0.009
Pe-nme(16:0/18:1(11Z))	Glycerophospholipid metabolism	732.56				**+**	7.6	0.04
Pe-nme2(16:1(9Z)/18:1(11Z))	Glycerophospholipid metabolism	744.56				**+**	3.6	0.01
Pe-nme(18:1(9Z)/18:3(9Z,12Z,15Z))	Glycerophospholipid metabolism	754.54				**+**	7.2	0.03
PA(20:3(8Z,11Z,14Z)/20:0)	Glycerophospholipid metabolism	755.56	**+**	2.8	0.008			
PC(14:0/20:0)	Glycerophospholipid metabolism	761.59				**+**	14.5	0.02
(2-{[3-(hexadecyloxy)-2-[(5E,8E,11E,14E,17E)-icosa-5,8,11,14,17-pentaenoyloxy]propyl phosphonato]oxy}ethyl)trimethylazanium	Unknown	766.57				**+**	3.9	0.03
PC(22:2(13Z,16Z)/14:1(9Z))	Glycerophospholipid metabolism	784.58				**+**	3.9	0.02
PC(22:4(7Z,10Z,13Z,16Z)/16:0)	Glycerophospholipid metabolism	810.60				**+**	2.7	0.001
PC(22:2(13Z,16Z)/16:1(9Z))	Glycerophospholipid metabolism	812.61				**+**	2.6	0.004
PC(22:6(4Z,7Z,10Z,13Z,16Z,19Z)/20:3(5Z,8Z,11Z))	Glycerophospholipid metabolism	856.58	**−**	2.8	0.01	**+**	3.0	0.002

(*§*) Putative Metabolites were identified through interpretation of tandem MS data. Feature identities were determined using a two-step approach described previously^76^: (1) search of public databases including METLIN, HMDB and Mass Bank using accurate mass and a mass error window of 10 ppm and (2) comparison of tandem MS data with available spectra for four important features followed by manual interpretation.

(*) The metabolic processes indicated in this column are the processes that most closely associated with the detected metabolites. This means that the detected metabolites may be an intermediate, a product or derivatives of intermediates or products of the mentioned processes. The metabolic processes were identified based on available information in HMDB, Kegg database and Lipidomics Gateway. The compounds for which we could not find any information are denoted as ‘Unknown’.

(+/−) Increase/Decrease.

## Discussion

This study highlights the metabolic changes induced by acute and short-term administration of THC in the gut of a murine model that has historically been used to demonstrate the positive health impacts of THC. To study these metabolic changes, comparative metabonomic profiling of fecal samples of THC-administered mice, and vector-administered mice were performed using a highly sensitive, accurate, and precise UPLC-ESI-QTOF-MS-based approach that has broad applications in metabonomic studies^51,52^.

With this, we have shown here that lipid metabolism, especially glycerophospholipid metabolism and fatty acid biosynthesis, is a key metabolic pathway targeted by THC following i.p. administration. Importantly, this pathway is intricately connected with several health disorders that are protected by THC; examples include Parkinson disease^53^, schizophrenia^54^, brain ischemia^55^, multiple sclerosis^56^ and cancer development^57,58^. Glycerophospholipids are precursors for several lipid mediators that, in collaboration with sphingolipids, participate in major signal transduction processes (see review by Farooqui *et al*.^59^) and along with sphingolipid metabolism, are functionally linked with several physiological and pathophysiological conditions that include but are not limited to pain, inflammation, metabolic syndrome, fibrosis, fertility, cancer and autoimmune and neurodegenerative disorders^60^. Others have also shown that glycerophospholipid and sphingolipid metabolism are the most significantly impaired pathways associated with the atherosclerosis progression^61,62^. Much of the protective role of cannabinoids on atherosclerotic coronary heart disease involves 15-lipoxygenase inhibitory activity, which in turn prevent lipid peroxidation, oxidative stress and atherosclerosis^63^. Based on our findings it is reasonable that THC-mediated protection against atherosclerosis and cardiovascular disorders can be linked to its regulatory effects on of glycerophospholipid and sphingolipid metabolism. We have conducted this study using adolescent mice to keep experimental consistency with our previous reports. While this age may seem irrelevant for some of the neurological disorders discussed above, we point out here that both young and adult mice have been used to understand the therapeutic impacts of THC on neuroinflammation and the associated health disorders such as autoimmune encephalitis^64^, Alzheimer’s disease^65^ and Parkinson’s disease^66^. Interestingly the increase in anti-inflammatory cytokine release in the brain of young mice can be mimicked by peripheral immune cells^67^.

Fecal metabolomics revealed an influence of THC on some additional major metabolic pathways which although connected with lipid metabolism, were not highlighted in our tissue metabolomic study. For example, a critical metabolite that feeds into Sphingolipid metabolism is L-serine, which is a metabolic output from the glycine, serine, and threonine metabolism^68^. The glycine, serine and threonine metabolic pathway feeds phosphatidylethanolamine to glycerophospholipid metabolism^69^. An upregulation of sphingolipid and glycerophospholipid metabolism, therefore, suggests an upregulation in Serine metabolism as well. Reduction of 2E-methyl glutaconic acid and tiglyglycine was observed upon 1X administration. These metabolites are often detected in human urine samples when the catabolism of branched-chain amino acids (BCAA) (especially isoleucine) is impaired^70,71^, suggesting that THC possibly influences BCAA catabolism. Emerging evidence supports the importance of BCAA catabolism in lowering the risk of type-2 diabetes^72^. While a previous study has shown that cannabidiol significantly reduces the incidence of diabetes in non-obese diabetic mice^73^, the relation between marijuana use and diabetes remains unclear. We also noted a significant reduction 2- methylene glutarate upon 1X administration suggesting downregulation of the metabolite flow from nicotinate degradation into pyruvate metabolism. This observation is in line with two recent reports that demonstrate a modulatory effect of cannabinoids and cannabinoid receptors on pyruvate (and energy) metabolism: (i) a report from Mendizabal-Zubiaga *et al*.^74^ which showed that expression of pyruvate metabolism genes increased in the striated muscle cells of CB1-knockout mice and, (ii) a report by Arrabal *et al*., which showed that pharmacological blockage CB1 was able to upregulate pyruvate metabolism enzymes^75^. It is hypothesized that such modulatory effects of THC and cannabinoids on energy metabolism may in part, contribute to their anti-tumor effects. Finally, an increased occurrence of two endogenous peptides upon 1X administration suggesting an activation of the endogenous opioid system. These peptides have receptors widely distributed in the central and peripheral nervous system and play key roles in immunity^76^, pain modulation^64^, emotion and stress response^65^, gut functioning^66^, neuroprotection with important implications in Parkinson’s disease^67^.

We point out here that, our study being untargeted in nature had three limitations that are typical for untargeted metabolomics: (1) a bias toward high-abundant metabolites (typical for LC-MS/MS), (2) the influence from exogenous metabolites such as those from gut microbiota (a common issue in fecal metabolome analysis) and (3) high-throughput analysis of samples without authentic standards, which although gives the advantage of the absence of *a priori* decisions, may lead to quantitative inaccuracy and in some cases compromise metabolite identity. The very high fold changes of enriched metabolites in fecal metabolite profiling could be either reflective of the influence of gut microbial metabolites while the differential abundance of certain metabolites only at one time point may indicate a bias toward high abundant metabolites. Regardless of these limitations, the strength of our study was our ability to conduct a comparative metabolomic examination of the fecal and intestinal tissue matrices (THC treated *versus* non-treated animals) in a holistic unbiased manner, which was helpful to test our central hypothesis and obtain a global understanding of how THC influences the host metabolic network. This provides us a scientific premise for developing new hypotheses for our future targeted metabolomic studies with diseased models. Such studies will focus on the cause-effect nature of the relationship between THC and the metabolic pathways identified in this study, under different pathophysiological conditions.

## Supplementary information

Supplementary Figures
