# Supplementary material for: Acute and short-term administrations of delta-9-tetrahydrocannabinol modulate major gut metabolomic regulatory pathways in C57BL/6 mice
Source: Sci Rep. 2019 Jul 19;9:10520. doi: 10.1038/s41598-019-46478-0 (PMC6642200; doi:10.1038/s41598-019-46478-0)
Supplement: Supplementary file 1 — Supplementary Figures [file 41598_2019_46478_MOESM1_ESM.pdf]

SUPPLEMENTARY INFOMATION

Acute and short-term administrations of delta-9-tetrahydrocannabinol modulate major gut metabolomic regulatory pathways in C57BL/6 mice

Megha Oza[‡, 1], William Becker[‡, 2], Phani M. Gummadidala[‡, 1], Travis Dias[‡, 1], Mayomi H. Omebeyinje[1], Li Chen[3], Chandrani Mitra[1], Rubaiya Jesmin[1], Paramita Chakraborty[4], Mathew Sajish[5], Lorne J. Hofseth[5], Koyeli Banerjee[6], Qian Wang[7], Peter D. R. Moeller[8], Mitzi Nagarkatti[2], Prakash Nagarkatti[2] and Anindya Chanda[*, 1]

Affiliations

[1] Environmental Health Sciences, Arnold School of Public Health, University of South Carolina, Columbia, SC, USA

[2] Department of Pathology, Microbiology, and Immunology, School of Medicine, University of South Carolina, Columbia, SC, USA

[3] Creative Proteomics Inc., Shirley, New York, USA

[4] Department of Statistics, University of South Carolina, Columbia, SC, USA

[5] Drug Discovery and Biomedical Sciences, College of Pharmacy, University of South Carolina, Columbia, SC, USA

[6] National Institute of Health, Bethesda, MD, USA

[7] Department of Chemistry and Biochemistry, University of South Carolina, Columbia, SC, USA

[8] National Ocean Service, Hollings Marine Laboratory, Charleston, SC, USA

‡ Co-primary authors
* Author for correspondence: achanda@mailbox.sc.edu

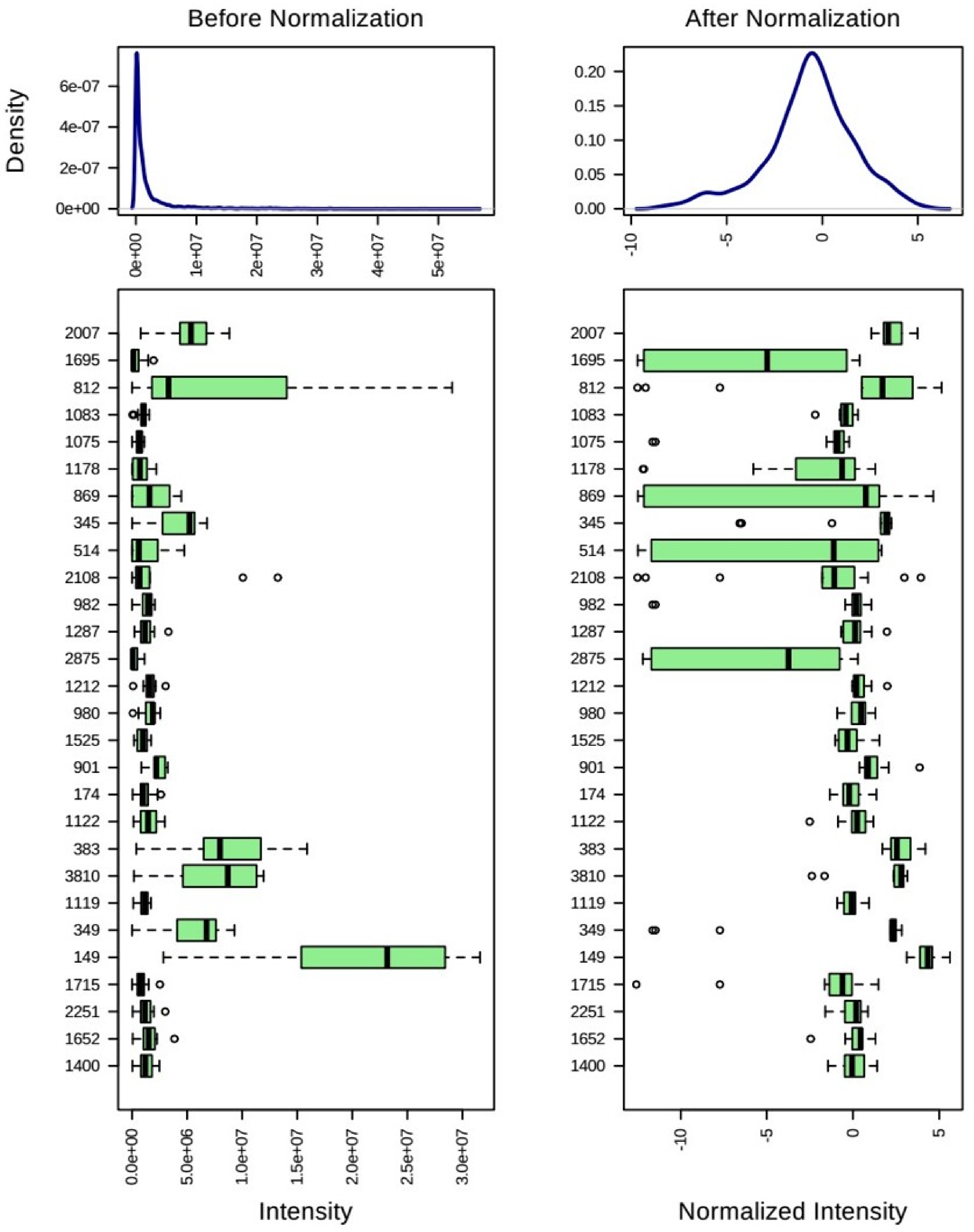

Before Normalization
After Normalization
Density
6e-07
4e-07
2e-07
0e+00
0e+00
1e+07
2e+07
3e+07
4e+07
5e+07
0.20
0.15
0.10
0.05
0.00
-10
-5
0
5
2007
1695
812
1083
1075
1178
869
345
514
2108
982
1287
2875
1212
980
1525
901
174
1122
383
3810
1119
349
149
1715
2251
1652
1400
0.0e+00
5.0e+06
1.0e+07
1.5e+07
2.0e+07
2.5e+07
3.0e+07
Intensity
Normalized Intensity

SI Fig 1. Normalization of ion peak intensities The raw data generated from Mass Hunter workstation was stored as a table with one sample per row and one variable (bin/peak/metabolite) per column. For comparability of metabolite profiles between samples, we implemented a three-category data normalization procedure that included: (a) sample specific normalization involving manual adjustment of concentrations based on biological inputs (i.e. volume, mass), (b) row-wise normalization, a normalization by the sample median for adjustment of differences among samples, and (c) a generalized log transformation of data (gLog2). Density plots and box plots before and after normalization are shown. The boxplots show at most 50 features due to space limit. The density plots are based on all samples.

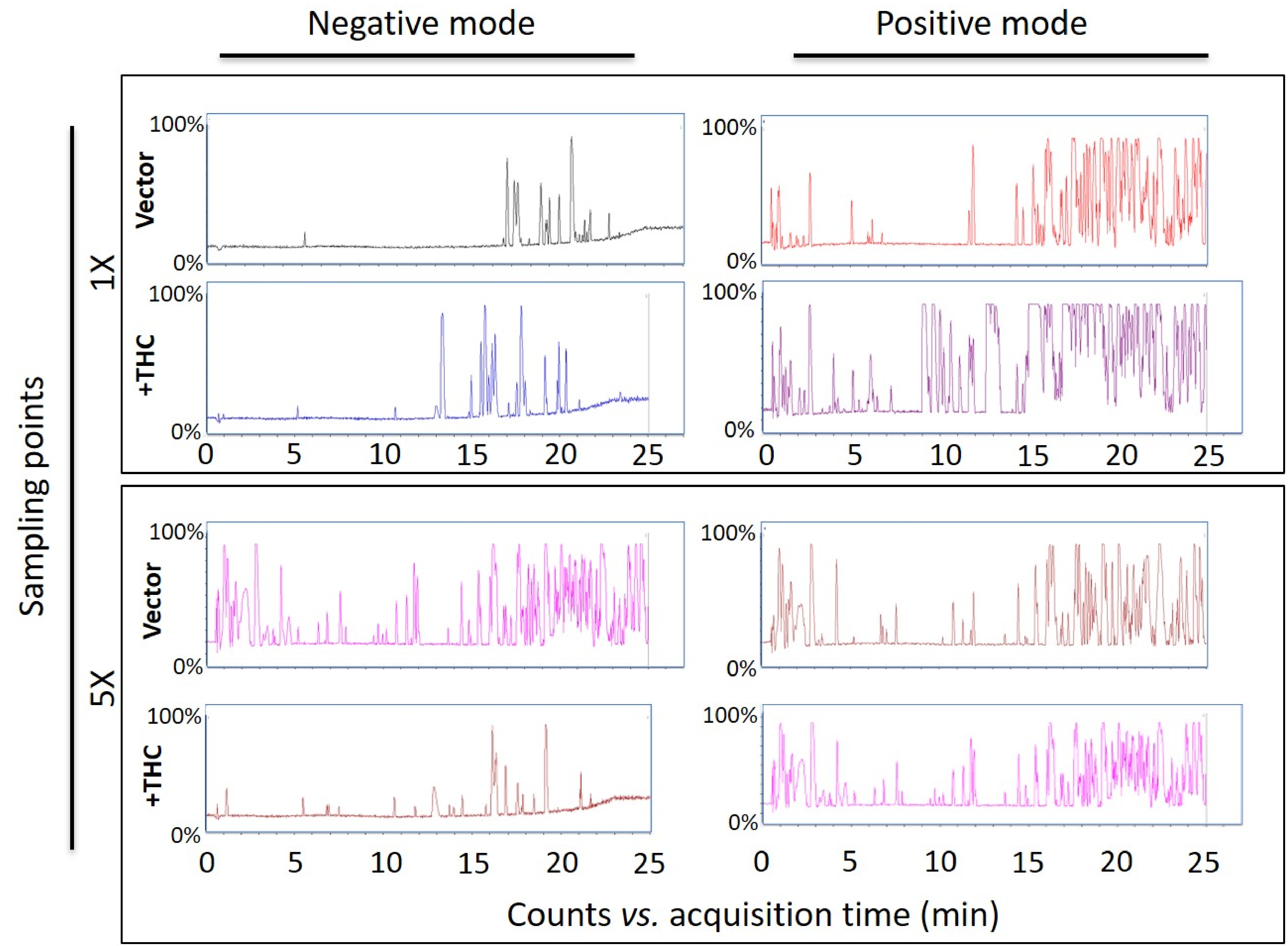

Negative mode
Positive mode
Sampling points
1X
5X
Vector
+THC
Vector
+THC
100%
0%
0
5
10
15
20
25
Counts vs. acquisition time (min)

SI Fig 2. Representative Base peak intensity (BPI) chromatograms of fecal extracts in positive and negative modes using UPLC-ESI-TOF-MS. Fecal samples (0.1g) were ultrasonically homogenized in 1mL cold methanol/water (1:1) in for 30mins followed by vortexing on cooled (4ºC) mixer for five mins. The homogenized samples were centrifuged (10000xg, 4ºC, 10 min). The supernatant (300 μL) was dried in a vacuum concentrator and re-dissolved in 150μL methanol/water (1:1) prior to analysis. Metabolites were separated from injected samples (5 μL aliquots) using Ultra Performance Liquid Chromatograph (1290 Infinity Binary LC System, Agilent Technologies, USA) and screened with ESI-MS (targeted MS/MS mode). The mobile phase consisted of 0.1% formic acid-water (solvent A) and 0.1% formic acid-acetonitrile (solvent B) with a gradient elution (0−1min, 95% A; 1−6min, 95−70% A; 6−20min, 70−5% A). The flow rate of the mobile phase was set at 0.5mL·min-1. The column temperature was maintained at 45°C, and the sample manager temperature was set at 4°C.

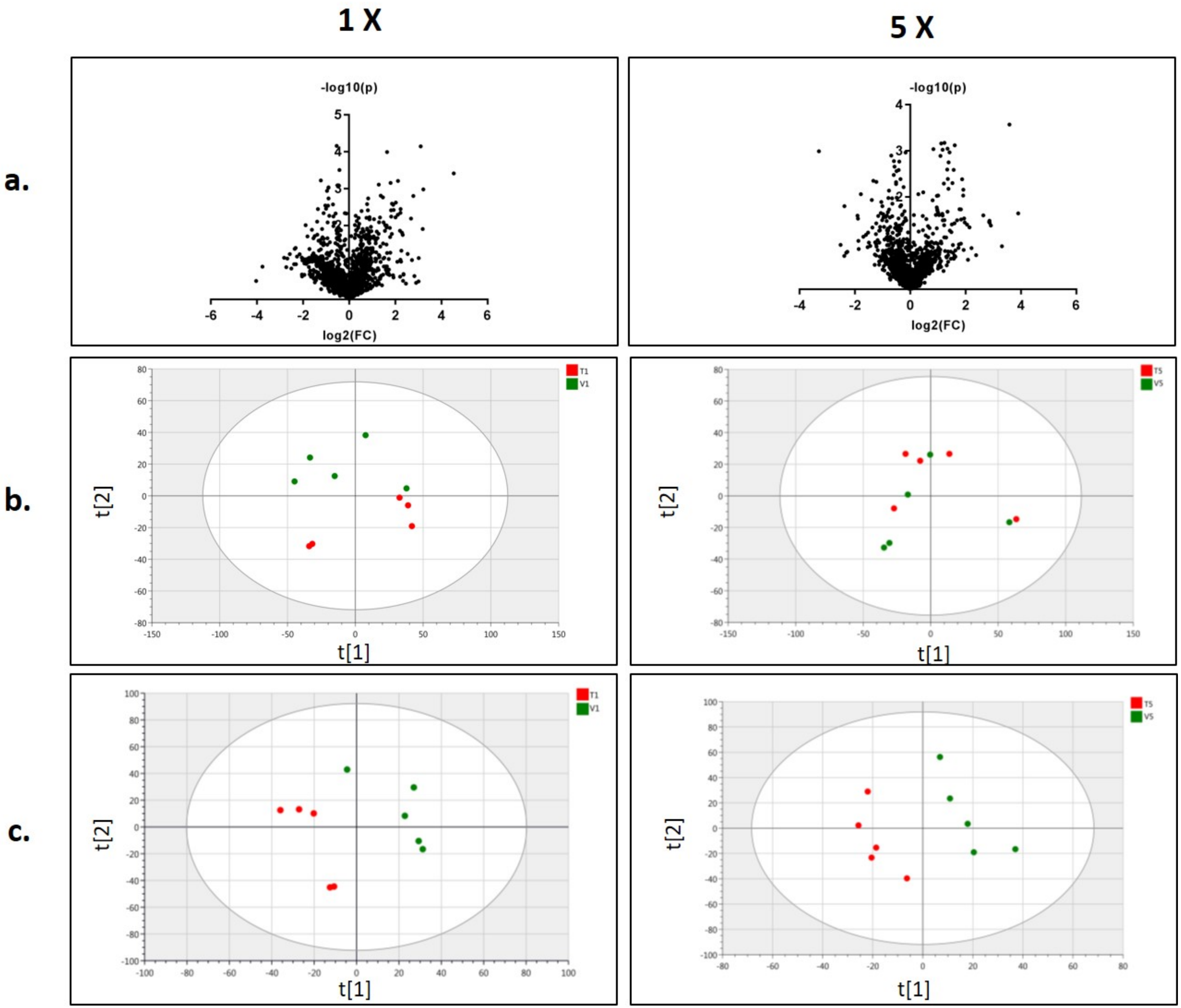
1 X
5 X
a.
b.
c.
-log10(p)
log2(FC)
t[1]
t[2]
T1
V1
T5
V5

SI Fig 3. Intestinal tissue metabolome comparisons between THC and vector administered mice. a) Volcano plots from 1X and 5X samples showing the metabolites with differential abundance between THC treated mice and the controls. These were selected based on fold change (X-axis) and p-value in (Y-axis). FC, fold change; p, p-value. Metabolites with fold change (increase/decrease) ≥ 2.0 and p-value ≤ 0.05 in THC administered mice compared to the vehicle controls and were selected for further characterization. b) PCA score scatter plots of 1X and 5X samples based on tissue metabolic profiling of THC (n = 5) and vehicle control (n = 5) mice c) PLS-DA score scatter plots of 1X and 5X samples based on tissue metabolic profiling of THC (n = 5) and vehicle control (n = 5) mice. T, THC administered mice, C, vehicle control mice.
